# Association between rs10757274 and rs2383206 SNPs as Genetic Risk Factors in Iranian Patients with Coronary Artery Disease 

**Published:** 2017-07

**Authors:** Seyed Ahmad Aleyasin, Tayebe Navidi, Saeed Davoudi

**Affiliations:** 1 *National Institute of Genetic Engineering and Biotechnology, Tehran, Iran.*; 2 *Tehran Heart Center, Tehran University of Medical Sciences, Tehran, Iran.*

**Keywords:** *Coronary artery disease*, *Chromosomes*, *Polymorphism, single nucleotide*

## Abstract

**Background:** There are only a few reports concerning the genetic risk factors for coronary artery disease (CAD). However, 2 polymorphisms of rs10757274 and rs2383206 on chromosome 9p21.3 have been shown recently to be associated with CAD in certain populations. This is the 1st study to investigate their validity and association with CAD in a sample of the Iranian population.

**Methods:** Genomic DNA was extracted from the peripheral blood of all participants, consisting of 111 cases with CAD and 100 normal controls with normal coronary angiographies. Genotyping of rs10757274 and rs2383206 was performed in the cases and controls using designed mismatch primers via the polymerase chain reaction-restriction fragment length polymorphism (PCR-RFLP) method.

**Results:** Statistical analysis presented a significant association between the rs10757274 GG (p value = 0.029, χ^2 ^= 7.078) and rs2383206 GG (p value = 0.036, χ^2^ =6.658) genotypes and CAD among the cases as compared with the normal controls. Haplotype analysis of rs10757274 and rs2383206 polymorphisms showed 43% GG/GG haplotype with a significant association with CAD (p value = 0.014, χ² = 6.058).

**Conclusion:** The results of this study provide an insight into the underlying molecular mechanism of CAD pathogenesis and pave the way for future functional studies on these variants.

## Introduction

Coronary artery disease (CAD), a progressive cardiovascular disorder, is the main cause of mortality worldwide.^1^ Numerous risk factors such as genetics, hypercholesterolemia, smoking, hypertension, and diabetes influence the development and severity of CAD.^2^ Genetic risk factors have been defined as important contributors to the pathogenesis of CAD. Recently, genome-wide association studies and meta-analyses on CAD and myocardial infarction have identified several genetic susceptibility loci including 9p21.3, 6q25.1, 1p13.3, 1q41, and 10q11.21. One of the strongest genetic linkages of CAD was detected on the chromosome 9p21.3 single nucleotide polymorphism (SNP) in different populations through genome-wide SNP association studies.^3^^-^^7^

Genome-wide SNP association studies in populations such as Caucasians from northern Europe, North America, and the German population have identified associations between CAD and SNPs such as rs1333049, rs1333040, rs2383207, rs10757278, rs10757274, and rs2383206 on chromosome 9p21. Among them, rs10757274 with higher incidence and overall mortality in CAD patients was suggested to have the strongest association with the disease.^8^^, ^^9^ These associations have also been reported in other ethnic groups such as Canadian, Danish, and American populations.^4^^, ^^10^^, ^^11^ Moreover, rs10757274 and rs2383206, located on chromosome 9p21.3, within a 20-kb distance of each other, are involved in A > G nucleotide change.^4^

The aim of this case-control study was to evaluate the association between the rs10757274 and rs2383206 variants and their related haplotypes and CAD in the Iranian population.

## Methods

In this study, a total of 211 participants, composed of 111 CAD cases and 100 normal controls aged between 45 and 50 years, were recruited. CAD cases were selected from individuals referred to Tehran Heart Center, Tehran University of Medical Sciences, Tehran, Iran. The cardiologists of the center consulted all the cases and normal controls based on their documented angiographies. The exclusion criteria were comprised of acute coronary syndrome, acute ischemic events, heart failure events, familial hypercholesterolemia, and previous cardiac surgery. Written informed consent was obtained from all the cases and controls. The study protocol was approved by the Ethics Committee of the National Institute for Genetic Engineering and Biotechnology, Tehran, Iran. 

 Normal control subjects in the same age range with no familial clinical CAD disorders were selected from the same geographical area as those of the cases. Premature CAD cases with diabetes or with high plasma cholesterol levels were excluded from this study in order to limit the confounding by the known major risk factors that strongly predispose individuals to CAD.

**Figure 1:**
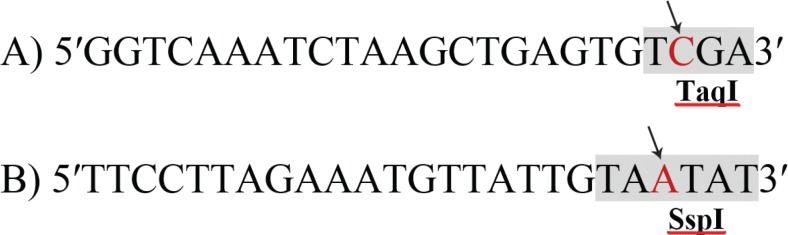
Mismatch forward primers sequences designed to amplify rs10757274 (A) and rs2383206 (B) polymerase chain reaction fragments by polymerase chain reaction. A substitution T to C at position 22 was considered for rs10757274 forward primer to create a restriction site for *TaqI* restriction enzyme (A). A substitution at position 22 G to A was considered to create a restriction site for the *SspI* restriction enzyme. Mismatched nucleotides are indicated by arrows.

Genomic DNA was extracted from peripheral blood lymphocytes using the standard salting-out method as described by Miller et al 1988.^12^ Since neither rs10757274 nor rs2383206 SNPs presented any restriction enzyme recognition site, two mismatch primers were designed to be use in RFLP (restriction fragment length polymorphism) method after polymerase chain reaction (PCR) amplification which called PCR-RFLP method These primers were designed by substitution of a mismatch base adjacent to their SNP sites using SNP Cutter software. Designing the rs10757274 forward mismatch primer by T to C substitution at base 22 provided a restriction site for the *TaqI* restriction enzyme. The forward mismatch primer for rs2383206 was by G to A substitution at base 22 which made a restriction site for *SspI* restriction enzyme (Figure 1). No sequence changes were applied to the reverse primers.

Each PCR reaction contained 18.3 μL of dH_2_O, 2.5μL of 10X *Taq* buffer, 1 μL of MgCl_2_, 0.5 μL of dNTP, 10 pmol of Forward and Reverse primers, 100 ng of genomic DNA, and 1.0 unit of *Taq* DNA polymerase (Fermentas, U.S.) in a total volume of 25 μL of PCR amplification for both rs10757274 and rs2383206. The PCR reaction was started with a single cycle of pre-denaturation at 94 ^°^C for 5 minutes, followed by 32 cycles of 94 ^°^C for 30 seconds, 62 ^°^C (rs10757274) and 57 ^°^C (rs2383206) for 1 minute, 72 ^°^C for 30 seconds, and a final extension at 72 ^°^C for 10 minutes. Genotyping of rs10757274 and rs2383206 were performed using *TaqI* and *SspI* restriction endonucleases digestion of their PCR products, respectively. The presence of the rs10757274 A allele in the 195-bp PCR product created a restriction site for the *TaqI* enzyme, detected by the appearance of 174-bp and 21-bp fragments. Similarly, the rs2383206 A allele in the 198-bp PCR product created a restriction site for the *SspI* enzyme, which resulted in 175-bp and 23-bp fragments. Restriction digestion reactions were performed in a total volume of 0.2 μL of the restriction enzyme (Fermentas, U.S.), 1 μL of 10X buffer, 1 μL of the PCR product, and 10 μL of dH_2_O. Digested fragments were separated by electrophoresis on a 12% non-denaturing polyacrylamide gel containing 1X TBE and were visualized through silver staining. Gel-purified PCR products were sent for sequencing (SEQ LAB, Germany) to confirm the genotyping and the subsequent Blast similarity search using the NCBI–Blast sequence similarity tool.

Allele frequencies were calculated for each genotype via the allele-counting method. The descriptive values are expressed as means ± standard deviations (SDs). Comparisons between the allele frequencies of the cases and those of the control groups were determined using the Pearson χ^2^ test using SNPstats software. Furthermore, 95% confidence intervals were considered significant covariates between the SNPs and CAD. A p value < 0.05 was considered statistically significant. Haplotype analysis was performed for the association between the rs10757274 and rs2383206 polymorphisms and CAD between 111 cases and 100 normal individuals using PHASE software, version 2.1.1.

## Results

There was no evidence of deviation from the Hardy–Weinberg equilibrium in either of the groups. The frequencies of the AA, AG, and GG genotypes for rs10757274 were 11%, 37%, and 51% in the CAD cases and 12%, 55%, and 34% in the controls, respectively. These frequencies for the rs2383206 genotypes were 9%, 55%, and 36% in the CAD cases and 9%, 53%, and 38% in the controls, correspondingly. The allele frequencies of the rs10757274 G allele and the A allele were calculated as 69% and 3% in the cases and 61% and 39% in the controls, respectively. The frequencies of the rs2383206 G allele and the A allele were 73% and 27% in the cases and 64% and 35% in the control group, respectively. The statistical analyses showed significant associations between rs10757274 (p < 0.029, χ^2 ^> 7.07) and rs2383206 (p < 0.036, χ^2^ > 6.65) and CAD in the cases as compared to the normal controls (Table 1).

The associations between rs10757274/rs2383206 AA/AA, AG/AA, AG/AG, GG/AG, GG/AG, and GG/GG were analyzed. The frequency of the GG/GG haplotype was low in the controls (27%) compared to that in the CAD cases (43%) (p < 0.014, χ² > 6.0) (Table 2).

**Table 1:** Comparison of the genotype and allele frequencies of the polymorphisms between case and control groups

Polymorphisms	Subjects	Genotype Frequencies % (n)			Allele Frequencies (%)		P value
rs10757274		AA	AG	GG	A	G	0.029
	Case (111)	11.7 (13)	36.9 (41)	51.4 (57)	30.2	69.8	
	Control (100)	12.0 (12)	54.0 (54)	34.0 (34)	39.0	61.0	
							
rs2383206		AA	AG	GG	A	G	0.036
	Case (111)	9.0 (10)	36.0 (40)	55.0 (61)	27.0	73.0	
	Control (100)	9.0 (9)	53.0 (53)	38.0 (38)	35.5	64.5	

**Table 2:** Comparison of the haplotype frequencies of rs10757274/rs2383206 between the CAD case and normal control groups.

Polymorphisms	Subjects	Haplotype Frequencies (%)					
rs10757274/rs2383206		GG/GG	GG/AG	AG/GG	AG/AG	AG/AA	GG/GG
	Case (111)	9.0 (10)	2.7 (3)	25.3 (28)	8.1 (9)	11.7 (13)	43.2 (43)
	Control (100)	5.0 (5)	6.0 (6)	41.0 (41)	6.0 (6)	10.0 (10)	27.0 (27)

CAD, Coronary artery disease.

## Discussion

Genome-wide association studies on CAD have identified a series of associated SNPs in an inter-genic region of chromosome 9p21.3 including rs10757274, rs2383206, rs10757278, and rs1333049. These SNPs have not been reported to be related to the other CAD risk factors and are known to act independently from the traditional CAD risk factors such as age, gender, obesity, smoking, hypertension, and hyperlipidemia. Among those SNPs on chromosome 9p21.3, rs10757274 and rs2383206 have shown a more significant association with CAD in recent studies.^3^^, ^^4^^, ^^8^^, ^^10^^, ^^13^^-^^17^ In the present study, the association between chromosome 9p21 SNPs rs10757274 and rs2383206 A to G and CAD was investigated in 111 CAD cases and 100 normal controls of the Iranian population. Our findings demonstrated a significant association between CAD and rs10757274 and rs2383206.

In our study, the frequency of the minor G allele of rs10757274 was 0.69 in the CAD cases, whereas a study on the African-American population reported a minimum frequency of 21% (p value = 0.004). A Pakistani study reported a maximum frequency of 0.86 in its case group.^18^ Middle frequency for the G allele was reported to range from 49% (p value = 0.025) in the American-Caucasian population to 62% in the Canarian race in Spain.^19^ The risk G allele frequency of rs10757274 in Iranians was more similar to that of Canarian, Irish, and Italian populations, respectively. This variant has been evaluated in different populations with significant associations with CAD, indicating its importance as a genetic risk factor for CAD. Apropos rs2383206, the frequency of the minor G allele was higher than that of the G allele of rs10757274 (73% vs. 69%) in the studied Iranian cases with CAD. The reported frequency in a different population was as minimum as 41% (p value = 0.0007) in the African-American population and the maximum frequency was reported as 59.8% in CAD cases in the Irish population.^20^ Middle frequency for the G allele was reported to range from 44% in the South Korean ethnicity to 55% in the German ethnicity. The risk G allele frequency for rs2383206 in Iranians was more similar to that in Irish and Italian populations.

Association analysis of the rs10757274/ rs2383206 GG/GG haplotype showed a significantly higher frequency among the cases than among the controls in the present study (p value = 0.014, χ^2^ = 6.058). This frequency was 55% and 48% in the Italian and South Korean populations, respectively.^6^^, ^^9^ The inter-genic locations of rs10757274 and rs2383206 on chromosome 9p21.3 are reported to influence the nearby *CDKN2A* and *CDKN2B* genes.^7^^, ^^13^^, ^^14^^, ^^21^ *CDKN2A* and *CDKN2B*, known as tumor suppressor genes, encode the inhibitors of cell-cycle kinases. Interestingly, the expressions of the *CDKN2A/CDKN2B/ANRIL* genes have been found to be associated with atherosclerosis severity through the risk haplotype GG/GG in CAD cases.^22^ An increased *CDKN2B* expression due to the presence of the risk haplotype GG/GG influences the expression of TGF-β, which is involved in the induction of atherosclerosis. 

Mapping analysis of the *ANRIL* gene, a non-coding RNA gene, by expression-sequence tags mapping technology has revealed its overlap with the CAD-related region on chromosome 9p21.3 and shown that its expression level is higher in the cells involved in atherosclerosis such as vascular cells, smooth muscle cells, and monocytes. Moreover, *ANRIL* is thought to be involved in the expression regulation of the neighboring protein-coding genes like *MTAP* and *CDKN2A*, *CDKN2B* and atherosclerosis development through vascular remodeling, thrombogenesis, and plaque stability.^23^ CAD is categorized in complex genetic disorders, and some diseases that predispose to CAD such as diabetes have been reported to be allied to rs10757274, rs2383206, rs10757278, and rs1333049 on chromosome 9p21.3. This may suggest this common genetic region as a risk factor for related complex diseases such as CAD and diabetes. 

One of the limitations in this study was the relatively small size of both case and control groups. Nevertheless, since a positive association was observed between both studied polymorphisms and CAD, it is not considered a major limitation. 

## Conclusion

Taken together, our findings represent further evidence that rs10757274 and rs2383206 are significantly associated with CAD in the Iranian population. To our knowledge, the present investigation is the first study to report the association between rs10757274 and rs2383206 and the risk of CAD in the Iranian population.

## Notes:

***This paper should be cited as:*** Aleyasin SA, Navidi T, Davoudi S. Association between rs10757274 and rs2383206 SNPs as Genetic Risk Factors in Iranian Patients with Coronary Artery Disease. J Teh Univ Heart Ctr 2017;12(3):114-118.
